# Antidepressant and anxiolytic potential of *Citrus reticulata* Blanco essential oil: a network pharmacology and animal model study

**DOI:** 10.3389/fphar.2024.1359427

**Published:** 2024-03-19

**Authors:** Nhi Phuc Khanh Nguyen, Ji-Hye Kwon, Min-Kyung Kim, Khoa Nguyen Tran, Ly Thi Huong Nguyen, In-Jun Yang

**Affiliations:** ^1^ Department of Physiology, Dongguk University College of Korean Medicine, Gyeongju, Republic of Korea; ^2^ Department of Pathology, University of Alabama at Birmingham, Birmingham, AL, United States

**Keywords:** Citrus reticulata Blanco, Chenpi, essential oil, depression, anxiety, rapid-acting effect, intranasal administration

## Abstract

**Background:**

*Citrus reticulata* Blanco essential oil (CBEO) has attracted increasing attention as a potential treatment for depression and anxiety in recent years. However, there is limited evidence regarding the active compounds responsible for its therapeutic effects. In addition, substantial amounts of CBEO and prolonged therapy are often required. This study aims to investigate the rapid acting antidepressant and anxiolytic effects of CBEO, identify the underlying composition as well as optimize its dosage and duration.

**Methods:**

CBEO composition was determined using gas chromatography–mass spectrometry (GC–MS), and the corresponding targets were obtained from the SwissTargetPrediction database. Depression-related targets were collected from DisGeNET, GeneCards, Therapeutic Target Database, and Online Mendelian Inheritance in Man. Subsequently, the overlap between CBEO and depression targets was utilized to build a network diagram depicting the relationship between the active ingredients and targets using Cytoscape software. The STRING database facilitated the construction of a protein–protein interaction network, and the Ma’ayan Laboratory Enrichment tool was employed for Gene Ontology (GO) enrichment, Kyoto Encyclopedia of Genes and Genomes (KEGG), and Wiki pathway analyses. Molecular docking was conducted using AutoDock Vina and Discovery Studio Visualizer. Topological analysis predicted the main antidepressant active ingredients in CBEO. A mixture of these compounds was prepared based on their relative GC–MS ratios. Tail suspension test, elevated plus maze, corticosterone-induced PC12 cells, and lipopolysaccharide (LPS)-induced BV2 cells were used to validate the antidepressant and anxiolytic potential of CBEO and CBEO’s main bioactive constituents.

**Results:**

CBEO contains 18 components that target 121 proteins. We identified 595 targets associated with depression; among them, 29 targets were located between essential oils and depression. Topological results revealed that linalool, p-cymene, α-terpinene, terpinen-4-ol, and α-terpineol were the major active compounds of CBEO in the management of depression. GO analysis identified G protein-coupled opioid receptor activity, phospholipase C-activating G protein-coupled receptor, and neuron projections that were mostly related to molecular functions, cellular components, and biological processes. Neuroactive ligand-receptor interactions, chemical carcinogenesis, and calcium signaling pathways were the major pathways identified in KEGG analysis. Molecular docking showed that the main bioactive ingredients of CBEO had favorable binding affinities for Protein-Protein Interaction’s hub proteins, including OPRM1, PTGS2, ESR1, SLC6A4, DRD2, and NR3C1. These five compounds were then mixed at 0.8:5:0.6:2:1 (w/w) ratio to form a CBEO antidepressant active compound mixture. An acute intranasal treatment of CBEO (25 mg/kg) only demonstrated an antidepressant effect, whereas the main bioactive compounds combination (12.5 mg/kg) illustrated both antidepressant and anxiolytic effects in mice. Linalool, p-cymene, and terpinene-4-ol exhibited neuroprotective and anti-neuroinflammation in the *in vitro* study, while these effects were not observed for α-terpinene and α-terpineol.

**Conclusion:**

Linalool, p-cymene, α-terpinene, terpinen-4-ol, and α-terpineol cymene might be mainly contributing to CBEO’s antidepressant effect by regulating neuroactive ligand-receptor interaction, neuron projection, and receptor signaling pathway. A mixture of these compounds showed rapid antidepressant potential via intranasal administration, which was comparable to that of CBEO. The mixture also exhibited an anxiolytic effect while not seen in CBEO.

## 1 Introduction

Depression is a common mental health condition characterized by enduring feelings of sadness, reduced interest in activities, and cognitive disruption. The global incidence of depression is increasing, affecting an estimated 28 million individuals annually (WHO, 2023). Currently, antidepressants, including serotonin-norepinephrine reuptake inhibitors (SNRIs) and selective serotonin reuptake inhibitors (SSRIs), are commonly prescribed in clinical settings. However, they often exhibit slow onset and limited efficacy, and can even induce suicidal tendencies (Rubino et al., 2007; Pompili et al., 2010). Therefore, there is an urgent need for novel antidepressants with improved efficacy and fewer adverse effects.

Essential oils have attracted increasing attention as potential sources of novel antidepressants in recent years. The main components of essential oils are aromatic molecules that can rapidly modulate psychological responses by directly stimulating the olfactory system. These tiny molecules can easily cross the blood-brain barrier to control the release and synthesis of depressive hormones and neurotransmitters (Cui et al., 2022). The results of an *in vivo* investigation have shown that the essential oil derived from *Paeonia lactiflora* Pall. alleviates corticosterone (CORT)-induced depression-like behavior through the activation of the PI3K/Akt/Nrf2 signaling pathway, leading to a reduction in neuronal apoptosis (Sun et al., 2022). *Aquilaria sinensis* (Lour.) Gilg in addition to *Aucklandia costus* Falc. reduced depression-related behavior in a rat model of chronic unpredictable mild stress by regulating the hypothalamic-pituitary-adrenal (HPA) axis and cholinergic and monoamine neurotransmitters (Li et al., 2020a). These data suggest the potential of essential oils in the management of depression.

Chenpi, the dried mature peel of *C. reticulata* Blanco, is used in traditional Chinese medicine prescriptions such as Tong-Xie-Yao-Fang. This herbal remedy showed a protective effect in mice exposed to chronic restraint stress by regulating the maturation of dendritic cells and the HPA axis (Jiang et al., 2023). Essential oil from *C. reticulata* (CBEO) is abundant in bioactive compounds such as flavonoids, terpenes, and coumarins. Pharmaceutical studies have shown that CBEO and its components have antioxidant, anti-inflammatory, anticancer, antibacterial, and antifungal properties (Dosoky and Setzer, 2018). According to a previous study, CBEO improves reserpine-induced depressive behavior, possibly by regulating the expression of brain-derived neurotrophic factors, glucocorticoid receptors, and 5-hydroxytryptamine-1A receptors (Tang et al., 2022). Another study revealed that CBEO possessed antidepressant activity by modulating the gut microbiota (Tsai et al., 2023). CBEO contains multiple components that exert its effects through various pathways. Many studies revealed that essential oil from *Citrus* spp. exhibited anxiolytic effects (Carvalho-Freitas and Costa, 2002; Ahsan et al., 2023). CBEO also demonstrated a tendency to alleviate anxiety; however, the effect was not statistically significant (Silveira et al., 2022). We hypothesize that standardizing CBEO by combining only its compound having therapeutic effects can improve its overall impact. To date, the compounds and main mechanisms underlying their therapeutic effects remain unclear. In addition, to exert a protective effect, long-term treatment (one to six weeks) and high concentration (100–500 mg/kg for oral administration) of CBEO are required. These limitations limit CBEO application in clinical practice and drug development.

To address the above issues, in this study, we used network pharmacology, a multi-layered and multi-faceted research strategy, to predict CBEO constituents and mechanisms of action that primarily contribute to its antidepressant potential. A mixture of CBEO’s main bioactive compounds was prepared at a ratio based on their relative content as determined by gas chromatography–mass spectrometry (GC–MS). The antidepressant- and anxiolytic-like effects of CBEO and its bioactive component mixture were validated by an *in-vivo* experiment in which intranasal administration was used to improve the dose and duration of treatment.

## 2 Materials and methods

### 2.1 Preparation of essential oil

Dried mature fruit peel of *C. reticulata* (500 g) was purchased from Omni Herb (Daegu, Korea) and validated by Professor In-Jun Yang (Dongguk University, Gyeongju, Republic of Korea) (voucher specimen 2022-A-41). The herb underwent a 4-h hydrodistillation process using a steam distillation solvent extraction apparatus. The obtained essential oil was weighed (yield 0.41% w/w) and stored at −20°C for further use.

### 2.2 Gas chromatography-mass spectrometric analysis

A GC-MS system (8890GC/5977MSD, Agilent, Santa Clara, CA, United States) was used to determine the chemical composition of CBEO. The chromatographic parameters are listed in Table 1. Unknown compounds were identified by comparing their mass spectra with those in the National Institute of Standards and Technology mass spectra database.

**TABLE 1:** Instrument conditions for gas chromatography–mass spectrometry analysis.

Item	Condition
Detector	MSD
Column	DB-5MS (30 m × 250 μm, 0.25 μm thickness)
Oven temperature	40 °C (5 min) → 2°C/min, 280 °C (10 min)
Helium gas flow (mL/min)	1
Injection volume (μL)	1
Split ratio	1:20
Inlet/detector temperature (°C)	250/280
Solvent delay (min)	3
Scan mode (amu)	50–500

### 2.3 Prediction of CBEO and depression-related targets

Putative targets of the CBEO ingredients were collected from SwissTargetPrediction (http://www.swisstargetprediction.ch/). “*Homo sapiens*” were chosen as species with probability >0 as the screening criteria. The Human Gene Databases, including DisGeNET (https://www.disgenet.org/), Online Mendelian Inheritance in Man (OMIM, https://omim.org/), GeneCards (https://www.genecards.org/), and the Therapeutic Target Database (TTD, http://bid.nus.edu.sg/group/cjttd/) were used to retrieve depression-linked targets. For the GeneCards and DisGeNet databases, the relevant score ≥10 and 0.3, respectively, was regarded as an inclusion criterion.

### 2.4 Construction of compound-target and protein-protein-interaction (PPI) network

The intersection of the CBEO and depression targets was input into Cytoscape Version 3.9.1 (https://cytpscape.org/) to visualize the compound-target network. Topological analysis was employed to obtain the “degree” and “betweenness centrality” of a network node. Compounds with these two values exceeding the mean were identified as the primary active components of CBEO for the treatment of depression.

The targets of CBEO for the treatment of depression were introduced into the STRING Database Version 12.0 (https://string-db.org/) with “Organism” was set as “*H. sapiens.*” The visualization procedure was conducted using Cytoscape and hub genes were chosen based on their high degrees of connectivity.

### 2.5 Gene function annotation and enrichment analysis

The target set of CBEO for depression treatment was introduced into Ma’ayan Laboratory Enrichment tool (https://maayanlab.cloud/Enrichr/) and the species was defined as “*H. sapiens*” for Gene Ontology (GO), Kyoto Encyclopedia of Genes and Genomes (KEGG) and Wiki pathway analyses. Bubble maps for enrichment analysis were generated using R language. By examining the outcomes of the GO, KEGG, and Wiki pathway analyses, the top pathways associated with depression were identified.

### 2.6 Molecular docking between active ingredients and hub genes

Molecular docking was performed between the selected active compounds and core proteins to confirm the predicted results using AutoDock Vina version 1.2.0 (https://vina.scripps.edu/). The docking results were visualized using the Discovery Studio Visualizer 2021 (https://discover.3ds.com/).

### 2.7 Animal experiments and treatments

Male ICR mice (5-week-old, 25–29 g) were purchased from Koatech Lab Animal, Inc. (Seoul, Korea). Mice were housed in groups (4 animals/cage; cage 35 × 18 × 12 cm) under a 12/12 h light-dark regime (22–23°C, 45%–50% humidity) with water and a standard pellet diet (5L79, PMI Nutrition, St Louis, MO, United States) available *ad libitum*. All experimental animal procedures were approved by the Institutional Animal Care and Use Committee of Dongguk University (IACUC-2023-18).

We used 3% of Tween 80 in saline as a vehicle. The compound mixture (MX) was prepared by mixing linalool, p-cymene, α-terpinene, terpinen-4-ol, and α-terpineol, the main bioactive compounds of CBEO, in ratios based on GC-MS results 0.8:5:0.6:2:1 (w/w). Previous studies investigated the antidepressant effects of essential oils and the compounds at doses of 10–80 mg/kg (Victoria et al., 2013; Duan et al., 2015) and 5–50 mg/kg (Melo et al., 2011; Han et al., 2013), respectively. Our previous study also showed that the rapid antidepressant effect of CBEO occurred at 25 mg/kg (Tran et al., 2023). These dose ranges were therefore applied in this study. In addition, memantine (MEM, 3 mg/kg, intraperitoneal) demonstrated an antidepressant effect hence it was employed as the positive control (Almeida et al., 2006a; Almeida et al., 2006b; Moriguchi et al., 2020; Alzarea et al., 2022).

The mice were randomly divided into the following five groups (n = 8 per group): CON (vehicle-treated group, intranasal, 10 µL), CBEO (25 mg/kg of CBEO, intranasal, 10 µL), MX_12.5 (12.5 mg/kg of CBEO bioactive compound mixture, intranasal, 10 µL), MX_6.25 (6.25 mg/kg of CBEO bioactive compound mixture, intranasal, 10 µL), and MEM (3 mg/kg, intraperitoneal, 200 µL). Behavioral tests were performed after 30 min of treatment as references (Han et al., 2013; Duan et al., 2015).

### 2.8 Tail suspension test (TST)

To measure antidepression-like effects, a TST was performed. The mouse was suspended by its tail using a 15-cm adhesive tape. During each 6-min trial, immobility time (defined as no movement of forelimbs) of each mouse was measured by the SMART v3.0 software (Panlab Harvard Apparatus, MA, United States).

### 2.9 Elevated plus-maze test (EPM)

Anxiety-related behavior was assessed using a black elevated plus maze (arm length, 29 cm; arm width, 5 cm; height above ground, 40 cm). The maze comprised two “open arms” and two “closed arms” surrounded by 14 cm high black walls (Tschenett et al., 2003; Bath et al., 2012). During each 5-min trial, the mouse was placed in the central area, facing one of the “closed arms,” and its activities, including total distance, mean speed, resting time, time and distance spent in the arms, were measured using Smart V3.0 software (Panlab Harvard Apparatus, MA, United States).

### 2.10 Toxicity evaluation

To investigate the safety of CBEO treatment in mice, the olfactory bulbs of mice were collected, washed with cold PBS, homogenized, and the levels of lactate dehydrogenase (LDH) were determined using an LDH kit in accordance with the manufacturer’s instructions (Abcam, Boston, United States).

### 2.11 Cell culture and treatments

PC12 cells were cultured in RPMI media supplemented with 10% FBS (Merck KGaA, Darmstadt, Germany) and 1% penicillin–streptomycin (Thermo Fisher Scientific, Waltham, MA, United States) at 37°C and 5% CO_2._ The cells were treated with linalool, p-cymene, α-terpinene, terpinen-4-ol, α-terpineol (0.1. 1, 10, 50, or 100 μM) with or without CORT (200 μM) for 24 h. The concentration of CORT was chosen based on our previous study (Tran et al., 2023).

BV2 cells were incubated in DMEM media supplemented with 10% FBS (Merck KGaA, Darmstadt, Germany) and 1% penicillin-streptomycin (Thermo Fisher Scientific, Waltham, MA, United States) at 37°C and 5% CO_2._ The cells were treated with linalool, p-cymene, α-terpinene, terpinen-4-ol, and α-terpineol (50 μM or 100 μM) with or without lipopolysaccharide (LPS, 1 μg/mL) for 24 h. Dexamethasone (DEX, 10 μM), an anti-inflammatory agent was used as a positive control (Oh et al., 2019).

### 2.12 Water-soluble tetrazolium salt (WST) and LDH assays

The effects of the treatments on the viability and LDH release of PC12 cells were examined using EZ-Cytox and EZ-LDH assay kits (DoGenBio, Seoul, Korea) following the manufacturer’s instructions. The absorbance was measured at 450 nm with a reference wavelength of 650 nm using an iMark microplate reader (Bio-Rad, Hercules, CA, United States).

### 2.13 Enzyme-linked immunosorbent assay (ELISA)

The levels of prostaglandin E2 (PGE2) in the BV2 cell supernatant after 24 h of treatment were analyzed using ELISA kits (LsBio, MA, United States) following the manufacturer’s protocols. The absorbance was measured at 450 nm with a reference wavelength of 650 nm using an iMark microplate reader (Bio-Rad, Hercules, CA, United States).

### 2.14 Western blot analysis

BV2 cells were lysed using the EzRIPA lysis kit (Atto, Tokyo, Japan). Proteins were separated using sodium dodecyl sulfate-polyacrylamide gel electrophoresis and transferred onto polyvinylidene fluoride membranes (Merck Millipore, Carrigtwohill, Ireland). Membranes were blocked with 5% skim milk and incubated with primary and secondary antibodies. The primary antibodies used included COX-2 (1:1,500, Santa Cruz Biotechnology, United States) and β-actin (1:10,000, Sigma-Aldrich). Protein bands were developed using enhanced chemiluminescence reagents and visualized using a ChemiDoc imager (Bio-Rad Laboratories). Band intensities were measured using GelPro v.3.1 software (Media Cybernetics, Rockville, MD, United States).

### 2.15 Statistical analysis

All experiments were performed independently at least three times. SPSS (version 29.0, https://www.ibm.com/) and GraphPad Prism (version 8.0; https://www.graphpad.com/) were used for statistical analysis and data visualization. The results are presented as means ± standard deviation, followed by statistical significance (One-way ANOVA with Dunnett’s *post hoc* test), defined as a *p*-value <0.05.

## 3 Results

### 3.1 Chemical composition of CBEO

**FIGURE 1:**
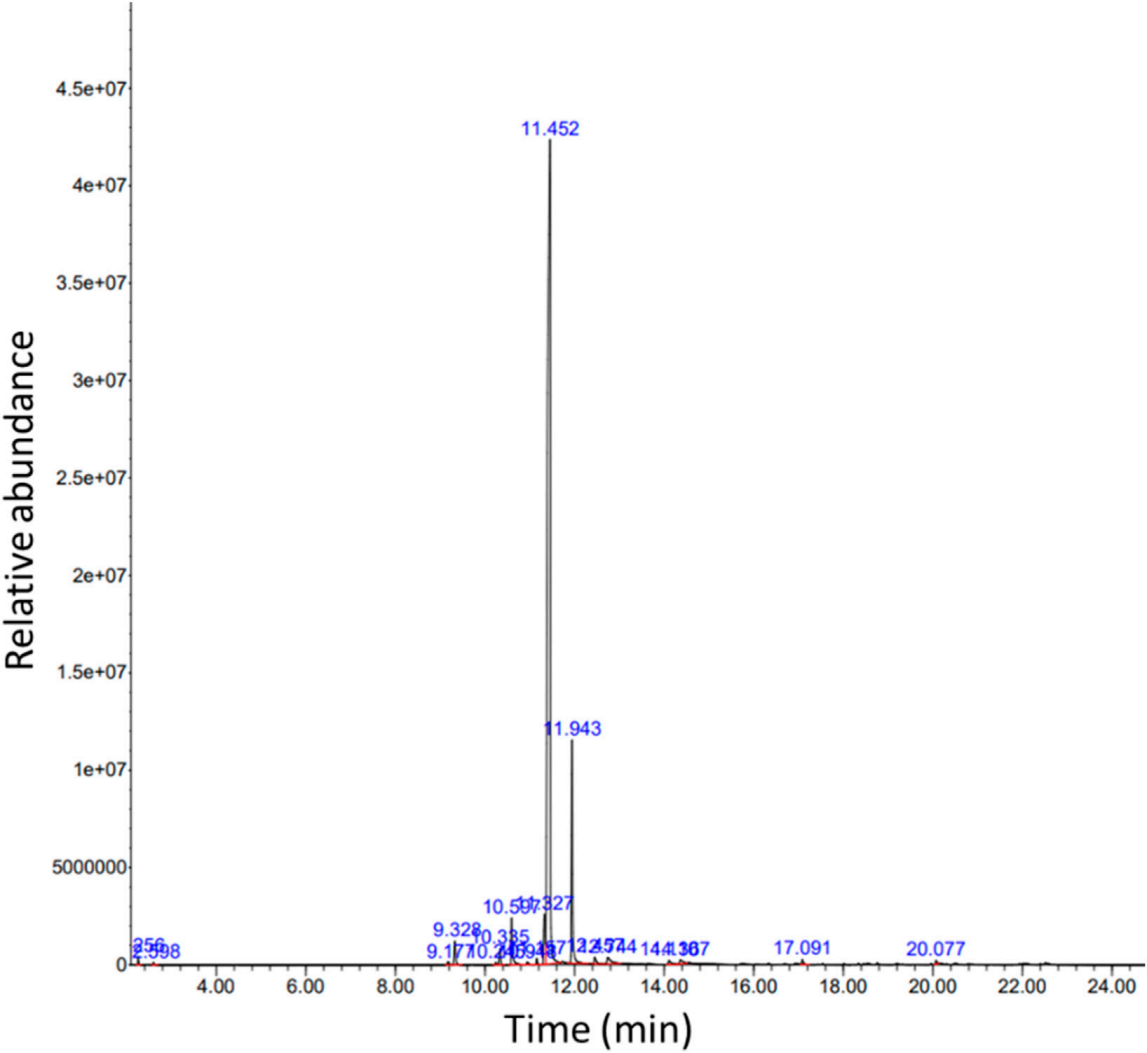
GC-MS chromatogram of CBEO. CBEO: *Citrus reticulata* Blanco essential oil; GC–MS: Gas chromatography–mass spectrometry.

The qualitative and quantitative compositions of CBEO were determined by GC–MS. The resulting data are shown in Figure 1 and Table 2. Eighteen compounds were identified in the essential oil, with D-limonene being the most abundant (80.7%), followed by γ-terpinene (8.8%), p-Cymene (2.5%), and β-myrcene (2.2%).

**TABLE 2:** Retention time and relative content of identified compounds of *Citrus reticulata* Blanco essential oil.

Compound	RT (min)	Relative content (%)
D-Limonene	11.452	80.7
γ-Terpinene	11.943	8.8
p-Cymene	11.327	2.5
β-Myrcene	10.597	2.2
α-Pinene	9.328	1.3
Linalool	12.544	1
(−)-β-Pinene	10.335	0.7
α-Terpineol	14.367	0.5
Isoterpinolene	12.477	0.4
Terpinen-4-ol	14.110	0.4
α-Terpinene	11.157	0.3
Timberol	20.077	0.3
Methylcyclopentane	2.560	0.2
α-Thujene	9.177	0.2
β-Elemene	17.091	0.2
Cyclohexane	2.598	0.1
Sabinene	10.243	0.1
α-Phellandrene	10.948	0.1

### 3.2 Prediction of potential targets of compounds and collection of targets for depression

**FIGURE 2:**
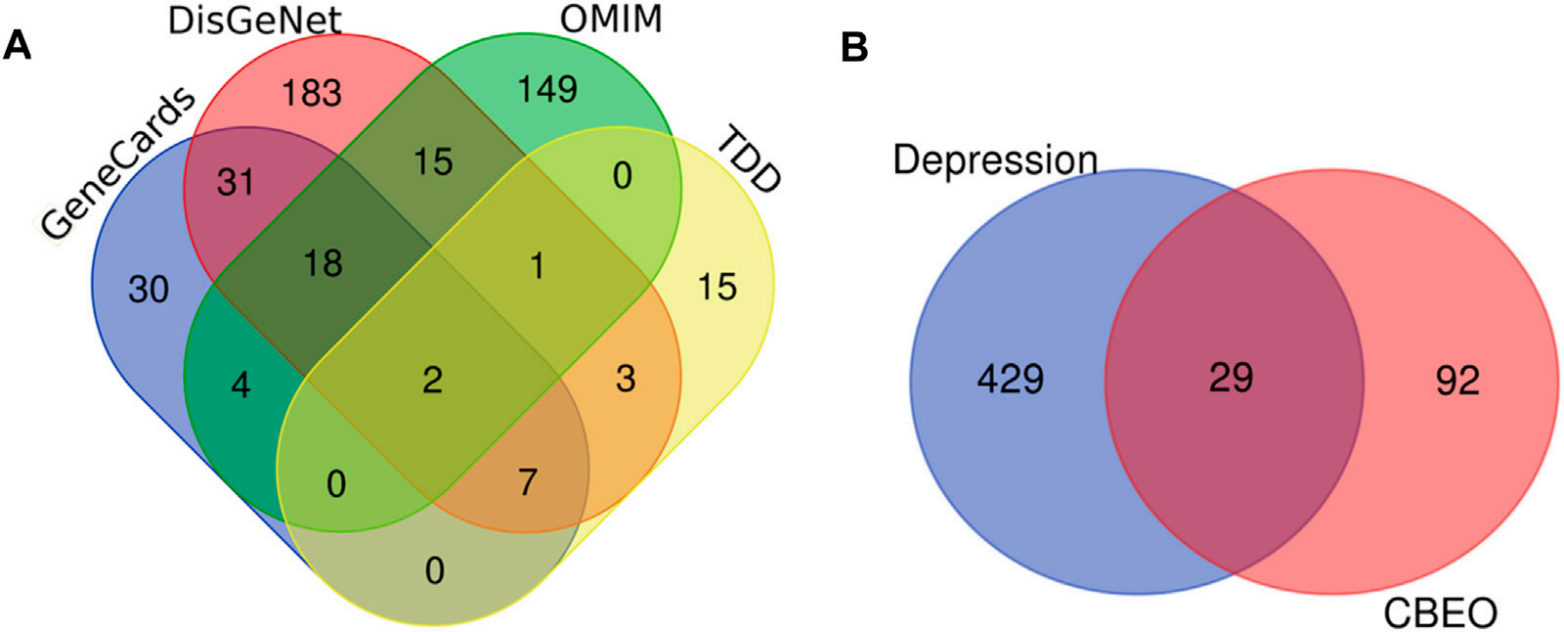
Target maps of CBEO and depression. **(A)** Depression targets in different disease databases. **(B)** Venn diagram of CBEO and depression targets. CBEO: *Citrus reticulata* Blanco essential oil.

SwissTargetPrediction predicted 237 potential targets for the 18 compounds identified using GC-MS. After eliminating the duplicate targets, 121 unique targets were identified (Supplementary Table S1). Data from the GeneCards, DisGeNET, TTD, and OMIM databases were gathered, leading to the identification of 82, 260, 29, and 376 depression-linked targets, respectively. Merging and removing duplicate targets resulted in 595 targets (Supplementary Table S2) (Figure 2A). Subsequent mapping of compound targets revealed 29 common targets shared by those associated with depression. These overlapping targets were ultimately identified as the target genes of CBEO for depression treatment (Figure 2B).

### 3.3 Compound-target and PPI network analysis

**FIGURE 3:**
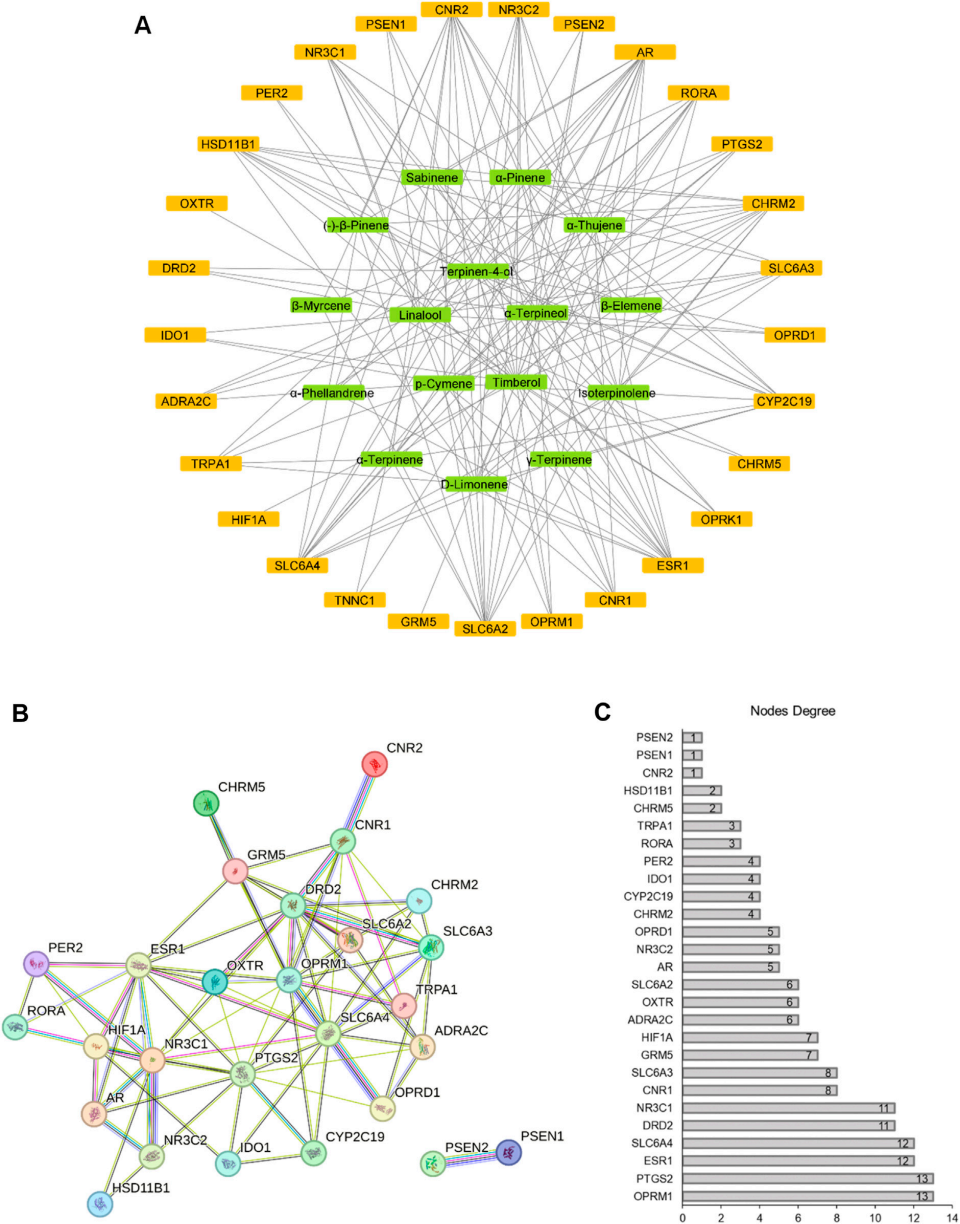
**(A)** Compound-target network. Green nodes represent chemical components, and orange nodes represent depression-related targets. **(B)** Protein–protein interaction (PPI) network. **(C)** Nodes degree of proteins.

We constructed a compound-target network incorporating 29 target genes associated with depression as potential antidepressant targets (Figure 3A). The network comprised 45 nodes and 178 edges, with 16 green nodes representing CBEO components, 29 orange nodes representing depression targets, and 178 edges representing the interactions between the CBEO components and depression targets. Examination of the network revealed instances in which the same active ingredient could act on multiple targets, and the same target could correspond to different chemical components. This observation underscores the multicomponent and multi-target nature of CBEO in the treatment of depression. Based on network topological parameters, the average “degree” and “betweenness centrality” of compound nodes were 11.125 and 0.049353, respectively (Table 3). Compounds with “degree” and “betweenness centrality” values exceeding the mean were identified, including linalool, p-cymene, α-terpinene, terpinen-4-ol, and α-terpineol, suggesting they may be the primary active ingredients of CBEO in treating depression.

**TABLE 3:** Topological features of compounds in the compound–target network.

Compound	Betweenness centrality	Degree
α-Thujene	0.03661	14
α-Pinene	0.03661	14
Sabinene	0.00722	8
(−)-β-Pinene	0.00722	8
β-Myrcene	0.00219	3
α-Phellandrene	0.00191	4
α-Terpinene	0.24505	19
p-Cymene	0.05287	13
D-Limonene	0.03661	14
γ-Terpinene	0.00805	8
Isoterpinolene	0.01357	10
Linalool	0.11298	17
Terpinen-4-ol	0.06587	15
α-Terpineol	0.15036	20
β-Elemene	0.00037	2
Timberol	0.01214	9
Mean	0.04935	11.125

The PPI network, highlighting potential connections between targets, consisted of 28 nodes and 82 edges, with an average node degree of 6, after excluding free genes (Figures 3B,C). Based on the degree values, the top six genes, OPRM1 (degree = 13), PTGS2 (degree = 13), ESR1 (degree = 12), SLC6A4 (degree = 12), DRD2 (degree = 11), and NR3C1 (degree = 11) were designated as hub genes.

### 3.4 Gene function annotation and enrichment analysis

**FIGURE 4:**
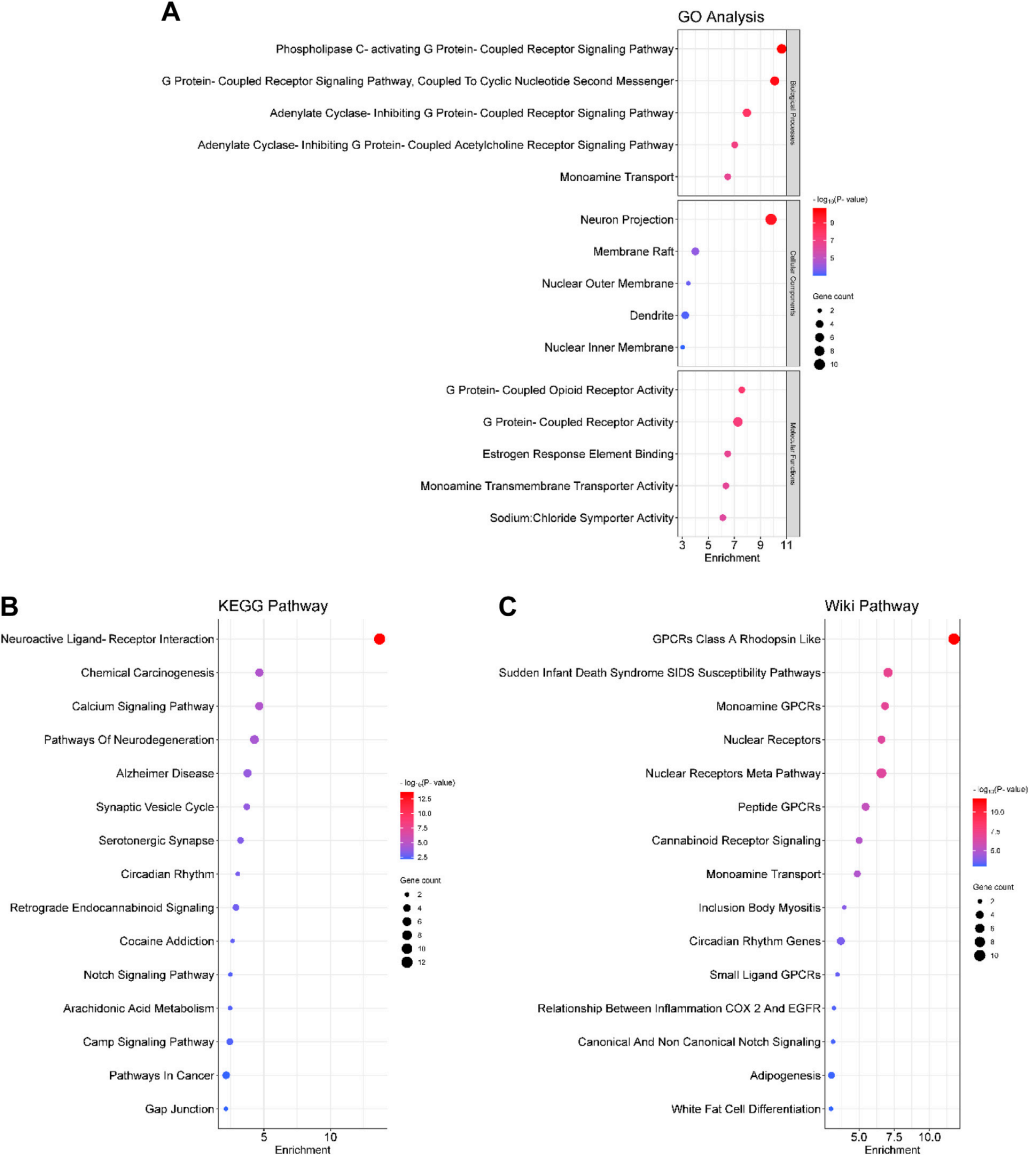
Bubble diagram for **(A)** GO, **(B)** KEGG and **(C)** Wiki enrichment analysis. The bubble size represents the number of enriched genes, and the bubble colour difference represents the significant magnitude of target gene enrichment. GO: Gene Ontology; KEGG: Kyoto Encyclopedia of Genes and Genomes.

We screened 20 biological processes, cellular components, and molecular functions by GO analysis. The top 3 biological process terms included “phospholipase C-activating G protein-coupled receptor signaling pathway,” “G protein-coupled receptor signaling pathway, coupled to cyclic nucleotide second messenger,” and “adenylate cyclase-inhibiting G protein-coupled receptor signaling pathway.” The top 3 GO cellular component terms comprised “neuron projection,” “membrane raft,” and “nuclear outer membrane”; and of molecular function terms are “G protein-coupled opioid receptor activity,” “G protein-coupled receptor activity,” and “estrogen response element binding” (Figure 4A).

To explore the signaling pathways related to the antidepressant-like effects of CBEO, we performed KEGG and Wiki enrichment analyses. The first 5 KEGG signal pathways involve “neuroactive ligand-receptor interaction,” “chemical carcinogenesis,” “calcium signaling pathway,” “pathways of neurodegeneration,” “Alzheimer disease” while those for Wiki pathways include “G protein-coupled receptor class A Rhodopsin like,” “sudden infant death syndrome susceptibility,” “monoamine G protein-coupled receptor,” “nuclear receptors,” and “nuclear receptors meta pathway.” In addition, Wiki enrichment results revealed that the relationship between COX-2 and EGFR inflammation might also contribute to the antidepressant effects of CBEO (Figures 4B,C).

### 3.5 Molecular docking between active ingredients and hub genes

**FIGURE 5:**
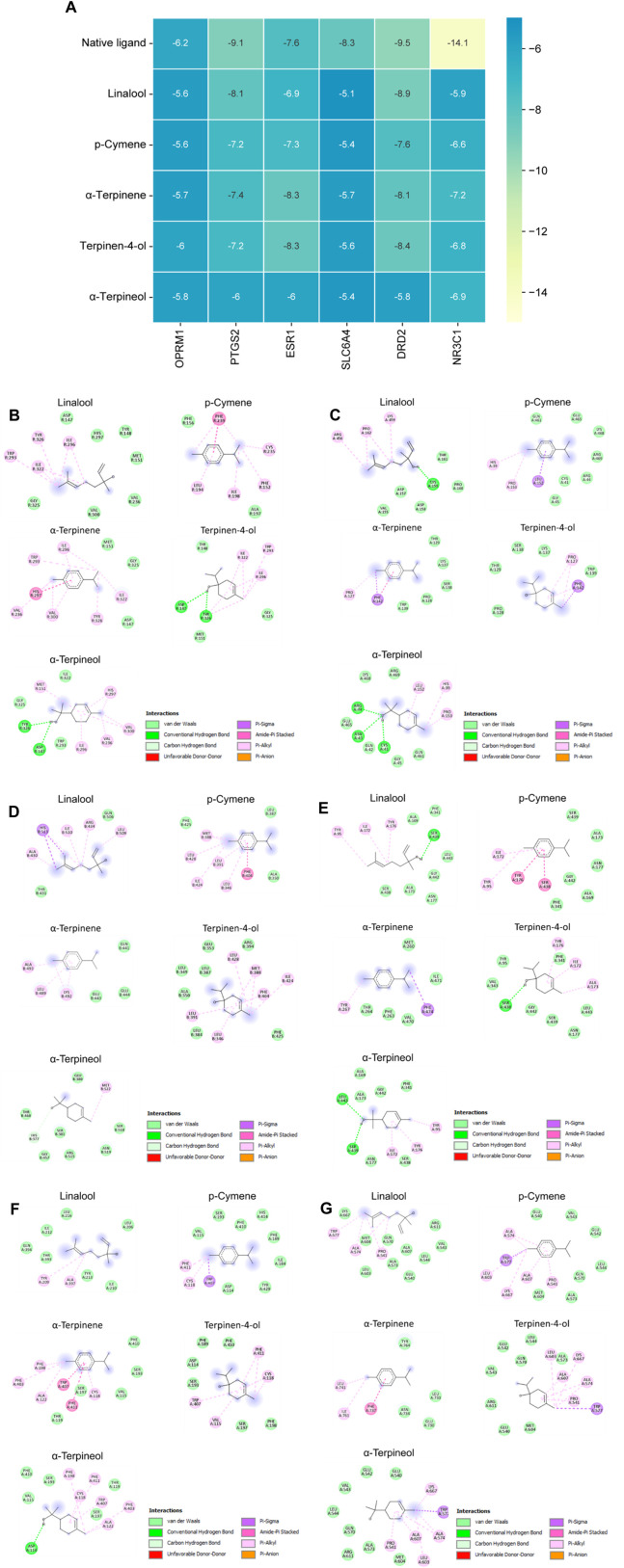
(Continued). **(A)** Heat map of molecular docking scores (kcal/mol^−1^). Native ligand represents the original ligand of the protein. 2D interactions of ligands with **(B)** OPRM1, **(C)** PTGS2, **(D)** ESR1, **(E)** SLC6A4, **(F)** DRD2 and **(G)** NR3C1.

We conducted molecular docking analysis on five active ingredients exhibiting node “degree” and “betweenness centrality” values higher than the average in the compound-target network. Additionally, we targeted OPRM1, PTGS2, ESR1, SLC6A4, DRD2, and NR3C1, which were the core proteins with the highest degrees in the PPI network. After molecular docking, the resulting data were subjected to a heatmap analysis, as shown in Figure 5A. Generally, lower energy levels indicate greater stability in the conformation of the ligand bound to the receptor, suggesting a higher likelihood of action. In our study, the binding energies of all active ingredients and core target proteins were <−5.0, signifying superior binding activity between CBEO active ingredients and core targets. The docking conformations of the compounds with the target proteins are shown in Figures 5B–G.

### 3.6 Rapid effect of CBEO and CBEO’s antidepressant active compounds mixture on depression-like and anxiety-like behaviors in mice

**FIGURE 6:**
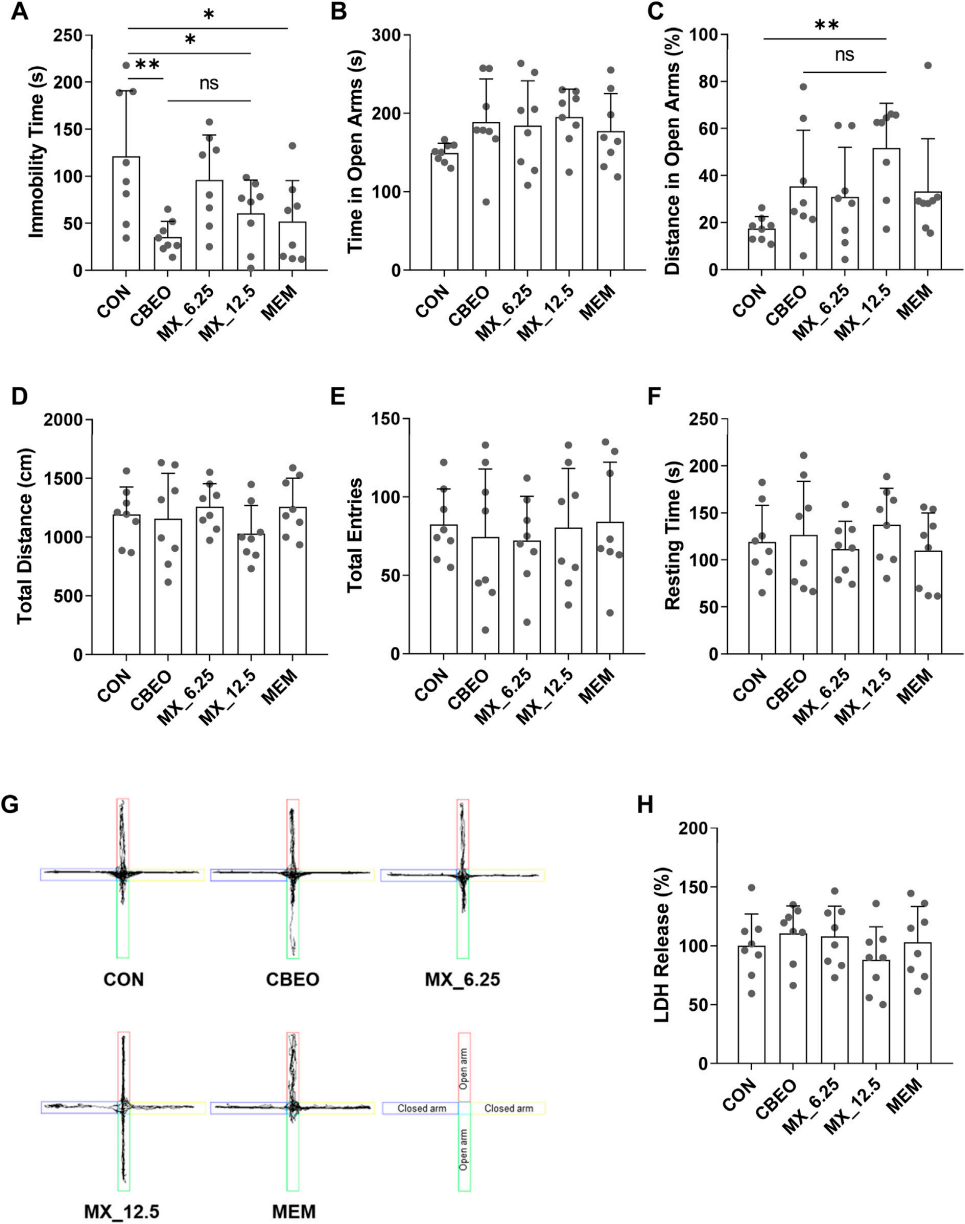
**(A)** The immobility time in a 6 min TST trial was recorded. The **(B)** time, **(C)** percentage of distance in open arms, **(D)** total distance, **(E)** total entries, **(F)** resting time and **(G)** traveling paths in a 5 min EPM trial were recorded. **(H)** LDH levels in olfactory bulbs were measured. Data are represented as means ± SDs (n = 8 per group). *p < 0.05, **p < 0.01 vs the CON group. ns: no significant; CON: control; MX_6.25 and MX_12.5: 6.25 and 12.5 mg/kg of bioactive compounds mixture; MEM: memantine; TST: tail suspension test; EPM: elevated plus-maze test.

To further validate the antidepressant effects of CBEO and its bioactive ingredient mixture, the behavior of mice after a single treatment was assessed using the TST and EPM. Following the topological and GC-MS results, the compounds were combined by mixing linalool, p-cymene, α-terpinene, terpinen-4-ol, and α-terpineol with the ratio 0.8:5:0.6:2:1 (w/w). ANOVA test indicated that there were significant differences among groups in terms of TST immobility time (F (4,35) = 4.618, *p* = 0.004) and the percentage of distance mice spent in the EPM open arms (F (4,35) = 3.145, *p* = 0.026) while none in the EPM exploration time (F (4,35) = 1.267, *p* = 0.301) (Figures 6A–C). Dunnett’s *post hoc* test showed that compared to the CON group, treatment with CBEO and MX_12.5 significantly reduced the immobility time in the TST (CBEO vs. CON, *p* = 0.002; MX_12.5 vs. CON, *p* = 0.041), exhibiting an effect similar to that of MEM. The results of the EPM demonstrated that MX_12.5 treatment significantly increased the percentage of distance mice spent in the open arms (*p* = 0.005). However, this effect was not observed in the EO-treated group. Administration of MX_6.25 resulted in no changes in mouse behavior during either the TST or EPM. There were no significant differences among groups which are related to total distance (F (4,35) = 0.976, *p* = 0.433), total entries (F (4,35) = 1.80, *p* = 0.947), and resting time (F (4,35) = 0.590, *p* = 0.672) in EPM test (Figures 6D–F), excluding the effect of the treatment on the locomotion of the mice. These results indicated that both CBEO and MX_12.5 exerted a rapid antidepressant effect, whereas a fast anxiolytic effect was only observed in MX_12.5-mice. In addition, the difference in the LDH levels in the olfactory bulb among groups was not significant (F (4, 35) = 0.835, *p* = 0.515), indicating the safety of CBEO and its compound mixture (Figure 6H).

### 3.7 Effects of CBEO compounds on CORT-induced neurotoxicity in PC12 cells

**FIGURE 7:**
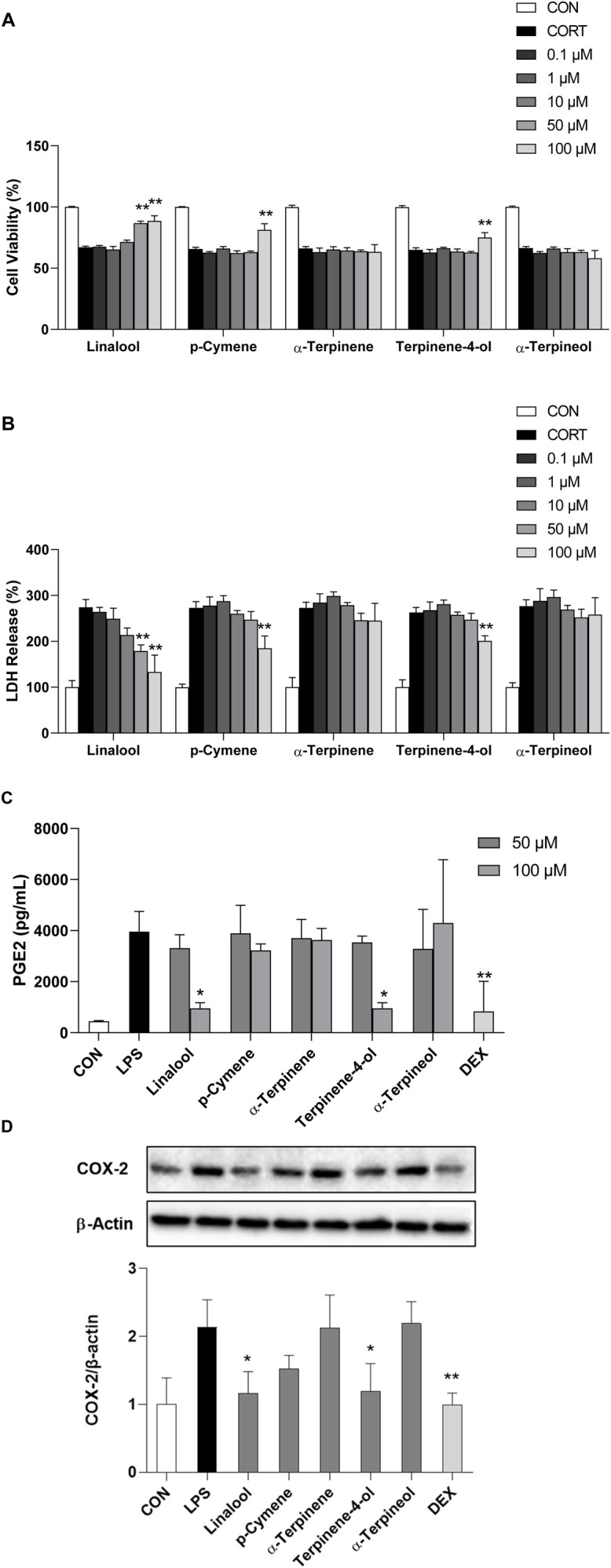
(Continued). Effect of CBEO compounds on CORT-induced neurotoxicity and LPS-induced neuroinflammation. **(A)** Cell viability and **(B)** LDH release in PC12 cells. **(C)** PGE2 level and **(D)** COX-2 expression in BV2 cells. Data are presented as means ± SDs (n = 3 per group). *p < 0.05, **p < 0.01 vs the CORT/LPS group. CORT: corticosterone; LPS: lipopolysaccharide; DEX: dexamethasone.

Figure 4B shows the potential involvement of the neurodegenerative pathway in the antidepressant effects of CBEO. This pathway was subsequently validated using CORT-induced PC12 cells treated with the major bioactive compounds of CBEO, namely, linalool, p-cymene, α-terpinene, terpinen-4-ol, and α-terpineol. To assess the potential toxicity of the CBEO compounds on PC12 cell viability, a WST assay was conducted. Notably, at doses of 0.1, 1, 10, 50, and 100 μM, none of the compounds demonstrated toxicity to PC12 cells (Supplementary Figure S1A), and these doses were chosen for subsequent experiments. ANOVA test revealed differences among groups in WST (linalool, F (6,14) = 98.639, *p* < 0.001; p-cymene, F (6,14) = 89.464, *p* < 0.001; α-terpinene, F (6,14) = 47.401, *p* < 0.001; α-terpinen-4-ol, F (6,14) = 80.516, *p* < 0.001; α-terpinenol, F (6,14) = 53.750, *p* < 0.001) and LDH (linalool, F (6,14) = 22.226, *p* < 0.001; p-cymene, F (6,14) = 32.999, *p* < 0.001; α-terpinene, F (6,14) = 26.802, *p* < 0.001; α-terpinen-4-ol, F (6,14) = 45.575, *p* < 0.001; α-terpinenol, F (6,14) = 22.932, *p* < 0.001) assays. Dunnett’s *post hoc* test demonstrated that 200 µM CORT treatment for 24 h induced cytotoxicity in PC12 cells, as demonstrated by a reduction in cell viability to 67% (*p* < 0.001) and a corresponding increase in LDH release to 270% (*p* < 0.001) compared to the control (CON) (Figures 7A,B). Pretreatment with linalool resulted in a dose-dependent increase in cell survival rate (*p* < 0.001) and a reduction in LDH levels (*p* < 0.01). However, p-cymene and terpinen-4-ol only at the highest dose (100 µM) demonstrated their reversed effects on the cell viability (p-cymene vs. CORT, *p* < 0.001; terpinen-4-ol, *p* = 0.002) and LDH levels (p-cymene vs. CORT, *p* = 0.001; terpinen-4-ol vs. CORT, *p* = 0.002), while no neuroprotective effect was observed for α-terpinene and α-terpineol.

### 3.8 Effects of essential oils on LPS-induced neuroinflammation in BV2 cells

As shown in Figure 4C, the antidepressant effects of CBEO may be linked to COX-2-related neuroinflammation. To test this hypothesis, an LPS-stimulated BV2 cell model was used. Initially, a WST assay was conducted to identify non-toxic doses of CBEO compounds in BV2 cells. Results showed that all doses (0.1, 1, 10, 50, and 100 µM) did not significantly reduce cell viability (Supplementary Figure S1B). Consequently, the highest doses, 50–100 μM and 100 µM were selected for ELISA and Western blot experiments, respectively. Among groups, there were significant differences related to the levels of PGE2 (F (12,26) = 5.912, *p* < 0.001) and COX-2 (F (7,16) = 6.87, *p* < 0.003). As depicted in Figures 7C,D, LPS (1 μg/mL) notably increased PGE2 concentration (*p* = 0.001) and COX-2 expression (*p* = 0.003) compared to those in the CON. Linalool and terpinen-4-ol at a dose of 100 µM mitigated the overproduction of PGE2 (linalool vs. LPS, *p* = 0.005; terpinen-4-ol vs. LPS, *p* = 0.005) and COX-2 (linalool vs. LPS, *p* = 0.009; terpinen-4-ol vs. LPS, *p* = 0.012), with effects comparable to DEX (10 µM). However, pretreatment with p-cymene, α-terpinene, and α-terpineol did not demonstrate anti-inflammatory effects in BV2 cells.

## 4 Discussion

This study explored the antidepressant potential of CBEO, which is the essential oil derived from the *C. reticulata* Blanco and investigated its composition responsible for that effect. Network pharmacology analysis revealed that the 18 compounds detected in CBEO by GC-MS targeted various genes and signaling pathways related to depression, emphasizing the multicompositional and multimechanistic characteristics of CBEO in the management of depression. The topological results showed that the main active components of CBEO against depression are linalool, p-cymene, α-terpinene, terpinen-4-ol, and α-terpineol. GO, KEGG, and Wiki enrichment analyses indicated the involvement of neurodegeneration and neuroinflammation in the effects of CBEO. The PPI analysis identified several key proteins that exhibited the highest degrees within the network, implying their potentially pivotal roles in mediating the antidepressant effect of CBEO. Notably, OPRM1, PTGS2, ESR1, SLC6A4, DRD2, and NR3C1 emerged as central nodes. OPRM1 is a receptor for endogenous opioids and its activation can lead to feelings of pleasure and euphoria, which are often disrupted in individuals with depression. Dysregulation of the opioid system is associated with anhedonia, a core symptom of depression (Pecina et al., 2019). PTGS2, also known as COX-2, is a part of the prostaglandin pathway that mediates the effects of inflammation on mood. Elevated levels of PTGS2 and proinflammatory prostaglandins have been observed in individuals with depression (Felger and Lotrich, 2013). ESR1 is a sex hormone receptor for estrogen. Estrogen has been shown to have neuroprotective and mood-regulatory effects (Hwang et al., 2021). Changes in estrogen levels can influence mood and contribute to a higher prevalence of depression in women (Kundakovic and Rocks, 2022). SLC6A4 encodes a serotonin transporter crucial for serotonin reuptake. Many antidepressant medications, such as SSRIs, target SLC6A4 to increase serotonin availability in the synaptic cleft, which can help alleviate depressive symptoms (Stevenson, 2018). DRD2 is a receptor for dopamine, a neurotransmitter involved in rewards, motivation, and pleasure. Dysregulation of the dopaminergic system has been implicated in mood disorders, particularly in the context of anhedonia and reduced motivation, which are common symptoms of depression (Delva and Stanwood, 2021). NR3C1 encodes the glucocorticoid receptor, which is sensitive to stress hormones such as cortisol. The stress response system is closely linked to depression, as prolonged or excessive stress can lead to dysregulation of the HPA axis and increased cortisol levels. NR3C1 is involved in regulating the body’s response to stress, and alterations in its function may contribute to the development and persistence of depressive symptoms in individuals exposed to chronic stress (Galbally et al., 2020). Molecular docking was conducted to validate the strength of the interactions between CBEO and these hub genes. Molecular docking results further revealed that all active ingredients of CBEO, including linalool, p-cymene, α-terpinene, terpinen-4-ol, α-terpineol, and key targets’ binding capabilities were <− 5.0. These data suggested that these five compounds mainly contributed to the antidepressant effects of CBEO.

We conducted an animal behavioral study to verify the antidepressant and anti-anxiety effects of CBEO using the TST and EPM. In the TST, depression is indicated by extended periods of immobility resulting from the animal’s futile efforts to escape an unavoidable situation (Can et al., 2012). The duration of immobility markedly decreased in animals administered a single dose of CBEO (25 mg/kg), indicating a rapid antidepressant effect. These results align with those of previous studies (Tang et al., 2022; Tsai et al., 2023), although those studies required a longer treatment duration (one to six weeks) and higher doses (100–500 mg/kg for oral administration), suggesting an outstanding advantage of intranasal administration. Compared with other routes, intranasal delivery has been prominent in drug development in recent years owing to its rapid effects and high bioavailability by crossing the blood–brain barrier directly and avoiding the first-pass effect (Shringarpure et al., 2021). The EPM was also conducted based on the innate conflict between a rodent’s tendency to explore new environments and their fear of open and exposed spaces (Walf and Frye, 2007). However, the anxiolytic effect was not observed in CBEO, only in the MX_12.5 group. Compared to the MX_12.5 mixture, CBEO contained lower levels of active antidepressant components. Certain inactive compounds in essential oils can function as solvents, leading to the dilution of the active components (Ogawa and Ito, 2016; 2019), which can explain the lack of anxiolytic effects of CBEO. Since a positive effect in behavioral experiments could be due to locomotion impairment, the integrity of the motor system was evaluated in treated mice. In our study, no significant changes in total distance, resting time, or total entries were seen among groups, excluding the possibility that the observed effect on anxiety and depression was due to a change in overall locomotion.

Although the antidepressant effects of CBEO have been reported, the compounds responsible for these effects remain unclear. Our network pharmacology predicted that linalool, p-cymene, α-terpinene, terpinen-4-ol, and α-terpineol were the main CBEO antidepressant components. The TST and EPM data confirmed that a single dose of the compound mixture (12.5 mg/kg) successfully reduced signs of depression and anxiety in mice, which is consistent with previous studies on the acute antidepressant effects of terpineol and linalool (Caputo et al., 2018; Vieira et al., 2020). However, compared with single-compound studies, our study employed a considerably lower dose (De Sousa et al., 2017). This can be explained by the synergistic effects of the components in the mixture (Dobrek and Głowacka, 2023). In our research, MEM served as a positive control. Several studies have consistently documented that MEM exhibits an antidepressant effect in animal models of depression, while the paradoxical effects, sometimes were reported in SSRI fluoxetine treatment (Gosselin et al., 2017; Krzystanek et al., 2021). Existing clinical trials have indicated that MEM displays antidepressant properties and is well-tolerated by patients, with mild side effects (Ferguson and Shingleton, 2007; Muhonen et al., 2008; Strzelecki et al., 2013). In addition to monotherapy, the combination of MEM with existing antidepressants has demonstrated an improvement in depressive symptoms of patients (Stevens et al., 2013; Amidfar et al., 2017; Lavretsky et al., 2020; Krzystanek et al., 2021). Moreover, we aimed to investigate the rapid antidepressant effects of CBEO and its compound mixture in this study. MEM has previously shown early-onset efficacy in individuals with depression (Ferguson and Shingleton, 2007; Amidfar et al., 2017) and also in animal model (Moryl et al., 1993; Kos and Popik, 2005; Almeida et al., 2006a; Almeida et al., 2006b; Réus et al., 2010; Tokita et al., 2012; Song et al., 2021), making it a suitable positive control for our research. For example, rapid antidepressant efficacy was demonstrated in mice administered memantine (3–10 mg/kg, i.p.) 30 min before the FST (Almeida et al., 2006b). Additionally, acute treatment with memantine (5–10 mg/kg, i.p.) given 60 min beforehand significantly reduced the immobility time of rats in the FST, showing a comparable effect to imipramine, a standard antidepressant (Réus et al., 2010). Moreover, when administered 40 min prior to the TST, memantine (2.5–15 mg/kg) induced a dose-dependent anti-immobility effect; notably, a significant reduction in immobility induced by 5 mg/kg of memantine persisted for up to 240 min post-administration (Kos and Popik, 2005). Until now, there have been many variations in the starting point of EPM. During the initiation of testing, the animal can be directed towards an open arm, or a closed arm, or positioned between an open and closed arm (at a 45° angle towards each arm) (Walf and Frye, 2007; Komada et al., 2008; Pawlak et al., 2012). Possible differences emerge when rodents face an open arm compared to facing a closed arm. Mice have a natural aversion to open spaces and prefer enclosed areas for safety. When first facing the closed arm, rodents might tend to run in that direction (Pellow et al., 1985). Consequently, they might perceive the open arms as more novel and scarier, resulting in hesitancy to explore open arms, and reducing time spent in these areas. However, the critical point is the same procedure should be used throughout a given EPM (Walf and Frye, 2007; Komada et al., 2008). Similarly, in our study, all of the mice, including the control and the treated groups, were placed facing the closed direction, reducing the effect of the starting point on the results of the experiment.

The neuroinflammatory response is a significant contributing factor to the development of depression. Studies have demonstrated that the activation of COX-2 leads to the production of PGE2, and increased activity in the PGE2 pathway effectively triggers depressive symptoms, likely by promoting oxidative stress (Chen et al., 2017; Song et al., 2019). The inhibition of COX-2/PGE2 has a neuroprotective impact on the dentate gyrus region, evidenced by the suppression of ROS in the mitochondria, upregulation of nuclear factor erythroid 2–related factor 2 (Nrf2) antioxidant function, and regulation of the pro-apoptotic and anti-apoptotic protein (Song et al., 2019). Previous research has suggested that COX-2/PGE2 pathway is also involved in the neuroprotective effects observed in SH-SY5Y and PC12 cells, mainly via controlling apoptosis and mitochondrial dysfunction (Geng et al., 2012; Li et al., 2020b). In this study, CORT-stimulated PC12 cells and LPS-stimulated BV2 cells were used to mimic neurodegeneration and neuroinflammation. Two possible mechanisms are involved in the antidepressant effects of CBEO (Figures 4B,C). Our findings indicated that besides alleviating cell death and LDH leakage, linalool and terpinene-4-ol possessed the ability to inhibit the excessive expression of COX-2 and the excessive production of PGE2. This implies that by suppressing inflammation, these two compounds may mitigate apoptosis and mitochondrial dysfunction, consequently contributing to their neuroprotective effects. In line with the *in vitro* results, linalool and terpinene-4-ol also demonstrated good docking scores with PTGS2. Compared with other compounds, terpinene-4-ol had a comparable (−7.2 kcal/mol) while linalool had a superior affinity (−8.1 kcal/mol) with PTGS2. A previous study explored the protective effect of p-cymene in H_2_O_2_-induced SH-SY5Y cells (Caputo et al., 2023). In our study, pretreatment with this compound can enhance cell viability and LDH over-release in CORT-induced PC12 cells, confirming the neuroprotective potential of p-cymene in different *in vitro* models of neurodegenerative diseases. In contrast, α-terpinene and α-terpineol did not exhibit protective effects in both neurotoxicity and neuroinflammation assays, suggesting the two compounds can exert their anti-depressant effect via other pathways. A previous study indicated that a mixture of terpineol isomers (α-terpineol—73%) showed an antidepressant-like effect through interaction with the DRD2 receptor (Vieira et al., 2020). Interestingly, our *in silico* study also predicted that α-terpineol has a good binding affinity with DRD2. To date, there have been limited studies on the antidepressant mechanism of α-terpinene, prompting a need for further investigations to address these gaps.

This study is subject to some limitations. To establish a comprehensive understanding of CBEO’s effects, it would be beneficial to investigate many doses of CBEO groups to observe the dose-effect relationship. Additionally, only male mice were used, and the influence of gender on the antidepressant activity of CBEO and its compound mixture has not been investigated in our study. Network pharmacology predicted many pathways contributing to CBEO’s therapeutic effect, however, this study only confirms two of them. Despite some studies to the contrary, MEM has not been shown to have antidepressant effect in humans or in animal models (Zarate et al., 2006; Smith et al., 2013; Papp et al., 2016). However, regulatory constraints prevent us from using the generally accepted rapid acting antidepressant, ketamine, as a positive control. Despite the problematic nature of memantine as a positive control, our dadirta show a clear effect of CBEO in an animal model of depression. Further investigations are needed to improve these limitations.

## 5 Conclusion

This study predicted that linalool, p-cymene, α-terpinene, terpinen-4-ol, and α-terpineol primarily contribute to CBEO’s anti-depressant effect, mainly via interactions with OPRM1, PTGS2, ESR1, SLC6A4, DRD2, and NR3C1, the six depression-related targets. The rapid therapeutic effects of CBEO may be related to neuroinflammation and neurodegeneration. Mixtures of the five bioactive compounds exerted both anti-depressant and anti-anxiety effects, suggesting that their efficacy in controlling psychiatric disorders was comparable to that of CBEO.

## Data Availability

The original contributions presented in the study are included in the article/Supplementary Material, further inquiries can be directed to the corresponding author.

## Abbreviations

SNRIs, Serotonin-norepinephrine reuptake inhibitors; SSRIs, Selective serotonin reuptake inhibitors; CORT, Corticosterone; HPA, Hypothalamic-pituitary-adrenal; CBEO, Essential oil from Citrus reticulata Blanco; GC–MS, Gas chromatography–mass spectrometry; OMIM, Online Mendelian Inheritance in Man; TTD, Therapeutic target database; PPI, Protein-protein-interaction; GO, Gene Ontology; KEGG, Kyoto Encyclopedia of Genes and Genomes; MEM, Memantine; CBEO, bioactive compound mixture; MX, LDH, Lactate dehydrogenase; DEX, Dexamethasone; ELISA, Enzyme-linked immunosorbent assay; PGE2, Prostaglandin E2; EPM, Elevated plus-maze test; TST, Tail suspension test; WST, Water-soluble tetrazolium salt.
