# Supplementary material for: Antidepressant and anxiolytic potential of Citrus reticulata Blanco essential oil: a network pharmacology and animal model study
Source: Front Pharmacol. 2024 Mar 19;15:1359427. doi: 10.3389/fphar.2024.1359427 (PMC10985240; doi:10.3389/fphar.2024.1359427)
Supplement: Supplementary file 1 [file Table1.DOCX]

**Table S1.** The 121 identified potential targets of CBEO.

| **No.** | **Gene symbol** | **Uniprot ID** |
| --- | --- | --- |
| 1 | ADORA1 | P30542 |
| 2 | ADORA2A | P29274 |
| 3 | ADORA3 | P0DMS8 |
| 4 | CNR2 | P34972 |
| 5 | PPARA | Q07869 |
| 6 | ACHE | P22303 |
| 7 | AR | P10275 |
| 8 | BCHE | P06276 |
| 9 | CHRM2 | P08172 |
| 10 | CYP19A1 | P11511 |
| 11 | CYP2C19 | P33261 |
| 12 | ESR1 | P03372 |
| 13 | FAAH | O00519 |
| 14 | PTPN1 | P18031 |
| 15 | SLC6A2 | P23975 |
| 16 | SLC6A4 | P31645 |
| 17 | TRPV1 | Q8NER1 |
| 18 | ACP1 | P24666 |
| 19 | ADRA2C | P18825 |
| 20 | AKR1B10 | O60218 |
| 21 | ATP12A | P54707 |
| 22 | CA1 | P00915 |
| 23 | CA2 | P00918 |
| 24 | CA4 | P22748 |
| 25 | CD81 | P60033 |
| 26 | CYP17A1 | P05093 |
| 27 | CYP51A1 | Q16850 |
| 28 | DRD2 | P14416 |
| 29 | ESR2 | Q92731 |
| 30 | FABP1 | P07148 |
| 31 | FABP3 | P05413 |
| 32 | FABP4 | P15090 |
| 33 | FABP5 | Q01469 |
| 34 | G6PD | P11413 |
| 35 | HMGCR | P04035 |
| 36 | HMOX1 | P09601 |
| 37 | HSD11B1 | P28845 |
| 38 | NPC1L1 | Q9UHC9 |
| 39 | NR1H3 | Q13133 |
| 40 | NR1I3 | Q14994 |
| 41 | NR3C1 | P04150 |
| 42 | NR3C2 | P08235 |
| 43 | PGR | P06401 |
| 44 | PLA2G1B | P04054 |
| 45 | PPARD | Q03181 |
| 46 | PTPN2 | P17706 |
| 47 | PTPN6 | P29350 |
| 48 | PTPRF | P10586 |
| 49 | RORA | P35398 |
| 50 | SCD | O00767 |
| 51 | SHBG | P04278 |
| 52 | SIGMAR1 | Q99720 |
| 53 | SLC6A3 | Q01959 |
| 54 | SQLE | Q14534 |
| 55 | SREBF2 | Q12772 |
| 56 | TRPM8 | Q7Z2W7 |
| 57 | TRPV3 | Q8NET8 |
| 58 | GLI1 | P08151 |
| 59 | GLI2 | P10070 |
| 60 | PLK1 | P53350 |
| 61 | ABHD6 | Q9BV23 |
| 62 | APH1A | Q96BI3 |
| 63 | APH1B | Q8WW43 |
| 64 | BACE1 | P56817 |
| 65 | CDC25A | P30304 |
| 66 | CDC25B | P30305 |
| 67 | CHRM1 | P11229 |
| 68 | CHRM3 | P20309 |
| 69 | CHRM4 | P08173 |
| 70 | CHRM5 | P08912 |
| 71 | CNR1 | P21554 |
| 72 | EPHX2 | P34913 |
| 73 | GABBR1 | Q9UBS5 |
| 74 | GCGR | P47871 |
| 75 | GPBAR1 | Q8TDU6 |
| 76 | GRM5 | P41594 |
| 77 | HIF1A | Q16665 |
| 78 | HSD17B2 | P37059 |
| 79 | ICMT | O60725 |
| 80 | IDO1 | P14902 |
| 81 | NCSTN | Q92542 |
| 82 | NR1H4 | Q96RI1 |
| 83 | OXTR | P30559 |
| 84 | PABPC1 | P11940 |
| 85 | PER2 | O15055 |
| 86 | PIK3CA | P42336 |
| 87 | PIK3CG | P48736 |
| 88 | POLA1 | P09884 |
| 89 | PRKCD | Q05655 |
| 90 | PSEN1 | P49768 |
| 91 | PSEN2 | P49810 |
| 92 | PSENEN | Q9NZ42 |
| 93 | PTGS2 | P35354 |
| 94 | SHH | Q15465 |
| 95 | SLC10A2 | Q12908 |
| 96 | UGT2B7 | P16662 |
| 97 | RBP4 | P02753 |
| 98 | ADRA1A | P35348 |
| 99 | HRH3 | Q9Y5N1 |
| 100 | HRH4 | Q9H3N8 |
| 101 | JAK1 | P23458 |
| 102 | JAK2 | O60674 |
| 103 | JAK3 | P52333 |
| 104 | KCNA5 | P22460 |
| 105 | LRRK2 | Q5S007 |
| 106 | OPRD1 | P41143 |
| 107 | OPRK1 | P41145 |
| 108 | OPRM1 | P35372 |
| 109 | PARP1 | P09874 |
| 110 | PTAFR | P25105 |
| 111 | SCN5A | Q14524 |
| 112 | SCN9A | Q15858 |
| 113 | TNNC1 | P63316 |
| 114 | TNNI3 | P19429 |
| 115 | TNNT2 | P45379 |
| 116 | TYK2 | P29597 |
| 117 | CYP2A6 | P11509 |
| 118 | PTGS1 | P23219 |
| 119 | TAAR1 | Q96RJ0 |
| 120 | TRPA1 | O75762 |
| 121 | RORC | P51449 |

**Table S2.** The 595 identified genes relevant to depression.

| **Gene symbol** | | | | | |
| --- | --- | --- | --- | --- | --- |
| A2M | AAGAB | AANAT | AARS1 | AARS2 | ABCA13 |
| ABCA7 | ABCB1 | ABCD1 | ABL1 | ACAD9 | ACE |
| ACKR1 | ADAMTS18 | ADCY1 | ADCY5 | ADCY7 | ADCY8 |
| ADCYAP1 | ADCYAP1R1 | ADH1B | ADH1C | ADRA2A | ADRA2C |
| ADRB1 | ADRB2 | AGO1 | AGT | AIM2 | AIP |
| AKT1 | ALB | ALG9 | ALK | ALPK3 | AMACR |
| ANKK1 | AP2S1 | AP3B1 | APAF1 | APOE | APOL2 |
| APOL4 | APP | APRT | AQP4 | AR | ARCN1 |
| ARHGEF10 | ARMC5 | ARNTL | ARRB2 | ARTN | ASMT |
| ATF3 | ATF4 | ATP1A2 | ATP1A3 | ATP2A2 | ATP6V0A2 |
| ATXN10 | ATXN2 | ATXN3 | ATXN8 | ATXN8OS | B2M |
| BAG1 | BCR | BDNF | BICC1 | BPNT1 | BRAF |
| BRCA1 | BSCL2 | BSN | BTK | C19ORF12 | C9orf72 |
| CACNA1A | CACNA1B | CACNA1C | CACNA1G | CALCA | CALM2 |
| CAMK2A | CAMK2B | CAMTA2 | CARS1 | CARTPT | CASP12 |
| CASP3 | CASR | CAV3 | CBS | CCDC8 | CD247 |
| CDH13 | CDKN2A | CEP85L | CHAT | CHCHD2 | CHI3L1 |
| CHRM2 | CHRM5 | CHRNA2 | CHRNA4 | CHRNA6 | CHRNB3 |
| CHRNG | CISD2 | CLCC1 | CLCN4 | CLN3 | CLN6 |
| CLOCK | CLRN1 | CMKLR1 | CNR1 | CNR2 | CNTF |
| COASY | COG4 | COL11A1 | COL2A1 | COL4A1 | COLEC10 |
| COLEC11 | COMT | COX15 | COX2 | CPLX1 | CPLX2 |
| CPOX | CREB1 | CRH | CRHBP | CRHR1 | CRHR2 |
| CRP | CRY1 | CRY2 | CSF1R | CSMD2 | CSNK1D |
| CTSF | CXCL8 | CYP2B6 | CYP2C19 | CYP2D6 | CYP3A4 |
| DAOA | DAOA-AS1 | DBH | DCTN1 | DEAF1 | DGCR8 |
| DGKB | DISC1 | DISC2 | DKK4 | DLG4 | DNAJC12 |
| DNAJC5 | DNMT1 | DOCK6 | DPP4 | DPYSL2 | DRD1 |
| DRD2 | DRD3 | DRD4 | DTNBP1 | DUSP1 | DUSP4 |
| DUSP6 | EBP | EDNRA | EED | EFHC1 | EFNB2 |
| EFNB3 | EGR3 | EIF2B4 | ELP1 | ELP4 | ENAM |
| ENPP1 | EPO | ERBB3 | ESR1 | EXT1 | F8 |
| FAM20C | FEV | FGD1 | FGF14 | FGF17 | FGF20 |
| FGFR1 | FGFR2 | FKBP5 | FLT4 | FMO3 | FMR1 |
| FOLH1 | FOS | FTO | FXN | FXR1 | FZD8 |
| G3BP1 | GABBR2 | GABRA1 | GABRA2 | GABRA3 | GABRA6 |
| GABRB3 | GAD1 | GAD2 | GAL | GAP43 | GAS1 |
| GBA1 | GCH1 | GDAP2 | GDNF | GFAP | GFRA1 |
| GGCX | GH1 | GJA5 | GLA | GLO1 | GLUL |
| GLYAT | GLYATL1 | GNAS | GNB3 | GPR3 | GPX1 |
| GRIA1 | GRIA2 | GRIA3 | GRID1 | GRID2 | GRIK3 |
| GRIN1 | GRIN2A | GRIN2B | GRK2 | GRM1 | GRM2 |
| GRM4 | GRM5 | GRM7 | GRN | GRPR | GSK3B |
| GSTM1 | GSTT1 | GTS | GYPE | HBA2 | HCN1 |
| HCRT | HCRTR1 | HDAC2 | HDAC4 | HDAC5 | HDAC6 |
| HDAC9 | HDC | HEXA | HIF1A | HLA-DQA1 | HLA-DQB1 |
| HLA-DRB1 | HMBS | HOMER1 | HP | HPCA | HPS1 |
| HRAS | HRH1 | HSD11B1 | HSPA1A | HTR1 | HTR1A |
| HTR1B | HTR1E | HTR2 | HTR2A | HTR2B | HTR2C |
| HTR3A | HTR3B | HTR4 | HTR7 | HTT | IARS1 |
| IDO1 | IFNA2 | IFNG | IFT140 | IGF1 | IL10 |
| IL18 | IL1A | IL1B | IL1RAPL1 | IL6 | IL6R |
| IMPA2 | INS | JAM2 | JPH3 | KANSL1 | KCND3 |
| KCNJ10 | KCNJ2 | KCNJ6 | KCNK2 | KCNT1 | KCTD17 |
| KIF17 | KIF21A | KMT2B | KMT2D | LDHA | LEP |
| LGI1 | LHPP | LIF | LMNB1 | LMTK3 | LRRC7 |
| LRRK | LRRTM1 | LRRTM2 | LTA4H | M6PR | MAGEL2 |
| MAOA | MAOB | MAPK1 | MAPK14 | MAPK3 | MAPK8 |
| MAPT | MASP1 | MBD1 | MC1R | MC4R | MCCC2 |
| MCHR1 | MECP2 | MED12 | MEN1 | MMACHC | MMP9 |
| MSTO1 | MTHFR | MTOR | MTR | MUC1 | MYF5 |
| MYH7 | MYL3 | MYLK2 | MYO7A | NCAM1 | NCDN |
| NCS1 | NEFM | NF1 | NGF | NGFR | NLGN4X |
| NMDAR1 | NOS1 | NOS2 | NOS3 | NOTCH2 | NOTCH3 |
| NPAS2 | NPS | NPSR1 | NPY | NR3C1 | NR3C2 |
| NR4A2 | NRG1 | NRN1 | NRXN1 | NTRK2 | NTS |
| OAS2 | OFD1 | OPHN1 | OPLAH | OPN4 | OPRD1 |
| OPRK1 | OPRM1 | OR7D4 | OTX2 | OVOL2 | OXT |
| OXTR | P2RX7 | PAH | PANK2 | PANX1 | PAX6 |
| PCDHAC2 | PCLO | PDCD1 | PDE11A | PDE1B | PDE2A |
| PDE4A | PDE4B | PDE4D | PDGFB | PDGFRB | PDYN |
| PDZD7 | PENK | PER2 | PER3 | PEX5L | PFKFB3 |
| PHEX | PHOX2B | PICK1 | PIK3R1 | PINK1 | PIP5K1C |
| PLA2G6 | PLEKHM2 | PMCH | PNOC | PNPT1 | POLG |
| POLG2 | POMC | PPP1R14A | PPP1R17 | PPP1R1B | PPP2R2B |
| PPP3CC | PPP3R1 | PPT1 | PRDX1 | PRICKLE2 | PRKACA |
| PRKAR1A | PRKCA | PRKCG | PRKCI | PRKCZ | PRL |
| PRNP | PRSS56 | PSEN1 | PSEN2 | PTGS2 | PTPA |
| PYY | RAC1 | RAD21 | RAP1A | RAP2A | RELN |
| REN | RIC3 | RIMS1 | RNF123 | RNU12 | RORA |
| RP1L1 | RRM2B | RTEL1 | RTN4R | S100A10 | S100B |
| SCN1A | SDHA | SDHB | SEPTIN9 | SERPINA6 | SFRP1 |
| SGCE | SHANK2 | SHANK3 | SHROOM4 | SIRT1 | SLC12A5 |
| SLC18A1 | SLC18A2 | SLC1A1 | SLC1A2 | SLC20A2 | SLC25A4 |
| SLC29A3 | SLC2A1 | SLC38A8 | SLC4A1 | SLC6A1 | SLC6A15 |
| SLC6A2 | SLC6A3 | SLC6A4 | SLC7A6OS | SLCO1C1 | SLITRK1 |
| SMIM30 | SMPD4 | SNAP25 | SNCA | SNCB | SOD1 |
| SOD2 | SORCS3 | SPAST | SRD5A1 | STK11 | STMN1 |
| STUB1 | STXBP1 | SYN1 | SYN2 | SYNJ1 | SYP |
| SYT3 | SYT7 | TAC1 | TACR1 | TACR2 | TACR3 |
| TAFAZZIN | TAP1 | TAS2R16 | TBC1D7 | TBL1XR1 | TBP |
| TBX1 | TBX19 | TCF4 | TDO2 | TET3 | TGM3 |
| TH | THAP1 | TIMELESS | TLE1 | TNF | TNFRSF1A |
| TNFRSF1B | TNNC1 | TOR1A | TP63 | TPH1 | TPH2 |
| TREX1 | TRH | TRNT1 | TRPA1 | TRRAP | TSC1 |
| TSC2 | TSPO | TTC19 | TTR | TWNK | TYMP |
| UBE2A | UCN | UQCRC1 | USH2A | USP8 | VAMP2 |
| VEGFA | VGF | VPS13A | WARS2 | WFS1 | WNT10A |
| WNT8A | WWC1 | XBP1 | XK | XPR1 | XYLT2 |
| ZEB2 |  |  |  |  |  |
