# Supplementary figures and images for: Antidepressant and anxiolytic potential of Citrus reticulata Blanco essential oil: a network pharmacology and animal model study
Source: Front Pharmacol. 2024 Mar 19;15:1359427. doi: 10.3389/fphar.2024.1359427 (PMC10985240; doi:10.3389/fphar.2024.1359427)

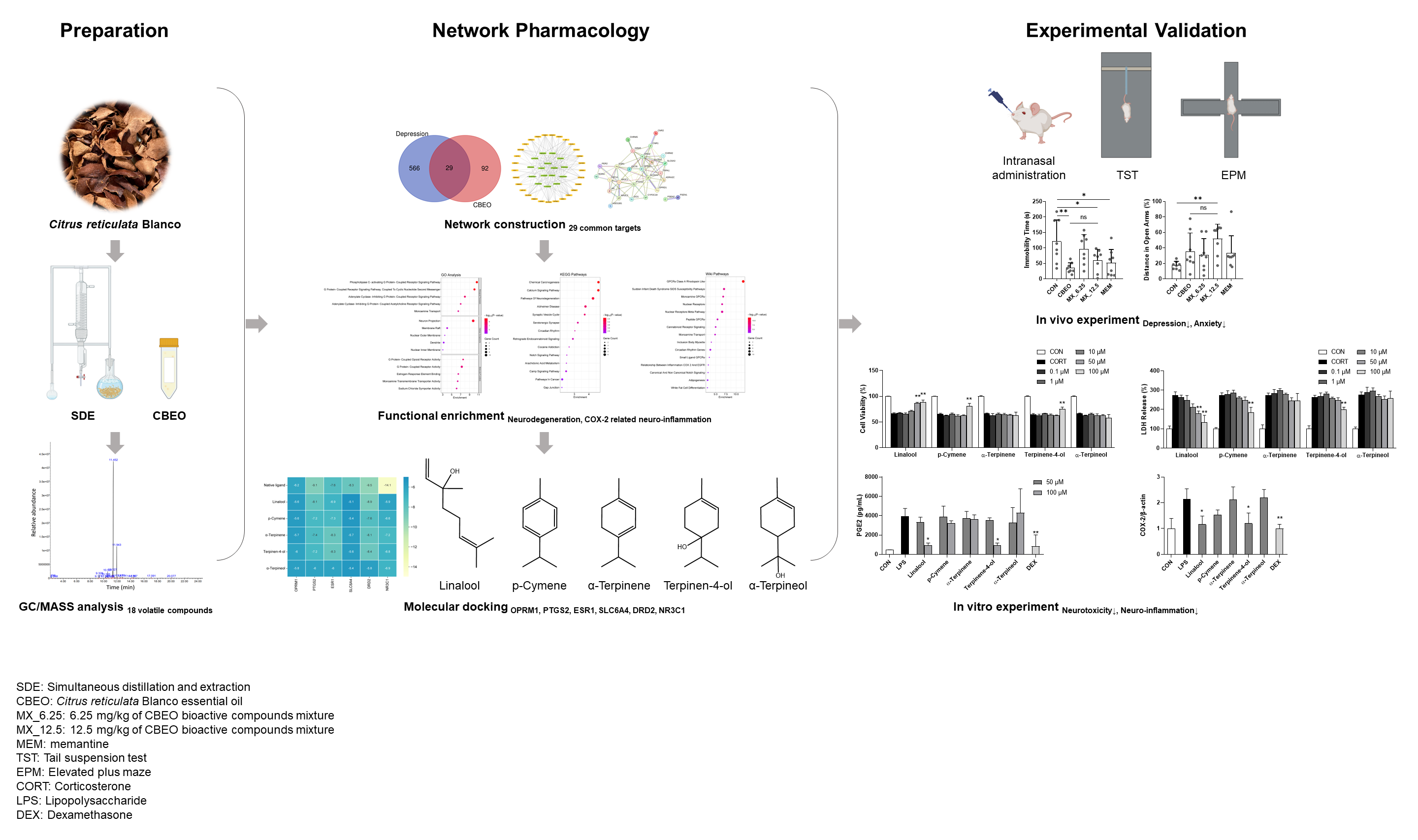

Supplement: Supplementary file 2 [file Image1.TIF]
